# The anatomy of prejudice during pandemic lockdowns: Evidence from a national panel study

**DOI:** 10.1371/journal.pone.0303845

**Published:** 2024-05-28

**Authors:** JohnMark Kempthorne, Kumar Yogeeswaran, Chris G. Sibley, Joseph A. Bulbulia

**Affiliations:** 1 School of Psychology, Victoria University of Wellington, Wellington, New Zealand; 2 School of Psychology, University of Canterbury, Christchurch, New Zealand; 3 School of Psychology, University of Auckland, Auckland, New Zealand; 4 Department of Linguistic and Cultural Evolution, Max Planck Institute for Evolutionary Anthropology, Leipzig, Germany; University of Padova, ITALY

## Abstract

During the early stages of the COVID-19 pandemic, there was a spike in the reporting of hate crimes (Human Rights Watch, 2020). However, the extent to which the pandemic affected prejudice across a general population—not merely among those disposed to hate crimes—remains unclear. Also unclear is the extent to which prejudice was restricted to specific minority groups associated with the virus, or whether prejudice spilled over to other minority groups. To address these questions, we use panel data collected from participants in a large national longitudinal (panel) study of New Zealanders before and during the early COVID-19 pandemic and systematically quantified social warmth ratings across a broad range of minority-groups (The New Zealand Attitudes and Values Study, N = 30,327, years 2018–2020). We discover reduced warmth toward Chinese, Asians (broadly defined), immigrants, Muslims, refugees, Indians, and the mentally ill. In absolute terms, warmth towards Chinese decreased the most (0.11 SD). Notably, changes in warmth were not detected toward NZ Europeans, Māori, Pacific Islanders, the overweight, or the elderly. Overall, these findings suggest that in New Zealand, pandemic prejudice may spread beyond minority groups associated with the virus to other groups perceived as non-prototypical of national identity.

## Introduction

Researchers postulate that, in addition to biological threats, deadly infectious diseases exacerbate social divisions [2, 3] Shortly after the SARS-CoV2 virus was announced in early 2020, reports suggested that Asians were being blamed for spreading the disease [4, 5] and targeted for hate crimes [6–9]. Yet whether attacks on Asians were the manifest expressions of more general and widespread attitudinal shifts remains unclear. Also unclear is whether prejudice was limited to those associated with the origin of the novel virus (China), or whether prejudice spilled over to other minority groups.

Some data suggested that the COVID-19 pandemic influenced prejudicial attitudes toward Asians in the United States [10]. Moreover, several studies found that prejudice was not limited to Asians. Pandemic prejudice was evident for immigrants in Greece, [11] Italy, [12] and a sample of 10 countries [13]. Pandemic prejudice was also evident for non-traditional women and gay people in Poland (i.e. “Sexual dissenters”) [14] and foreign residents in South Korea [15]. Across these studies, the factors associated with prejudice varied, and included COVID-19 concern (10 countries and Italy), COVID-19 prevalence (South Korea), lockdowns (Greece), COVID-19 outbreak (Poland), and contracting COVID-19 (US).

Although preliminary evidence suggests the pandemic fueled prejudice towards Asians and other minority groups, particularly ‘foreigners’, much remains unclear. Most studies lack clearly resolved presentative time-series data. Only one study (in Poland [14]) was nationally representative among key demographics. With only repeated cross-sectional data and unrepresentative samples, the observed associations might not generalize. Moreover, several studies that use time-series data do not leverage baseline measures for prejudice within individuals, increasing scope for bias in the estimation of effects (see: Method).

Additionally, the effect of the pandemic on prejudice is far from clear. While some studies report no clear evidence that the COVID-19 pandemic amplified prejudice, several studies suggest that the COVID-19 pandemic may have reduced prejudice by augmenting social solidarity. For instance, data from the Netherlands show a decline in disease concerns over the course of two years (from 2020 to 2022), but attitudes toward immigrants did not concurrently decline [16]. Likewise, fear of COVID-19 did not predict prejudice toward immigrants in data from five European countries in 2020 [17]. Whereas after COVID-19 started to spread, attitudes toward immigrants were more favorable among Californians [18] and attitudes toward asylum-seekers were more positive in a cohort of German men; [19] and in tweets from Turkey, negative sentiment toward refugees started to decline in association with quarantine-like restrictions in movement [20]. Unfortunately, evidence for reduced prejudice is also compromised by a combination of poorly resolved time-series data, selection bias from non-representative samples, and a limited bandwidth of prejudice measures from which to evaluate the scope of the pandemic prejudice spill-over.

Ideally, the study of pandemics would be informed by nationally representative panel studies. Panel data can be used to infer causality by providing data that resemble a natural experiment. Measures from the year before the pandemic provide appropriate baseline measures both for demography and pre-pandemic prejudice–measures that could not be affected by events that unfolded in the following year when the virus emerged. Because the attack of the virus was random with respect to data collection, the assignment mechanism to the pre or post pandemic condition in the year of the pandemic emulates a randomized controlled intervention. Furthermore, including prejudice measures toward a range of groups allows us to evaluate the relative effect-sizes of prejudice to minority groups who vary both in their association with the virus itself and in levels of perceived “foreignness.” Unfortunately, we are not aware of any previous national longitudinal (panel) study that investigates country-wide prejudice in the wake of COVID-19. Here, we attempt to fill this gap by combining panel data from a nationally representative longitudinal panel survey, the New Zealand Attitudes and Values Study (NZAVS) with systematic methods for identifying causal effects. By assessing attitudes toward multiple social groups during each wave, the combination of NZAVS data and rigorous causal methods may clarify whether prejudice toward Asians was general across a national population, and whether such prejudice extended to other minority groups.

## Method

### Sample

The New Zealand Attitudes and Values Study (NZAVS) is an annual longitudinal national probability panel study of social attitudes, personality, ideology and health outcomes. The NZAVS began in 2009 and is curated by Professor Chris Sibley. It includes questionnaire responses from more 72,000 New Zealand residents. The study includes researchers from many New Zealand universities, including the University of Auckland, Victoria University of Wellington, the University of Canterbury, the University of Otago, and Waikato University. Because the survey asks the same people to respond each year, it can track subtle change in attitudes and values over time. The NZAVS is university-based, not-for-profit and independent of political or corporate funding.

The NZAVS has ethical approval from the University of Auckland Human Participants Ethics Committee, reference number: UAHPEC22576. More information on the NZAVS is available at: www.nzavs.auckland.ac.nz.

Here, we use data from NZAVS Times 10 (2018–2019) and 11 (2019–2020). NZAVS sampling procedures for Time 10 and Time 11 are available in **S1** and **S2 Appendices**, respectively.

### New Zealand’s COVID-19 timeline

**Fig 1:**
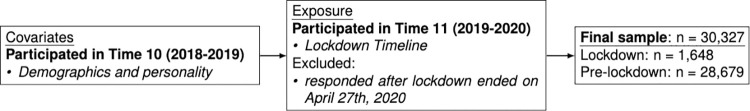
Criteria for filtering participants.

We use New Zealand’s 2020 COVID-19 timeline to identify pre/post Pandemic conditions for our causal comparisons (see **S3 Appendix**). The timeline of these events with respect to NZAVS data collection Time 11 can be found in **Fig 11** in S2 **Appendix**. The community transmission phase provides a natural period in which to demarcate the “treated” and “untreated” groups for comparison. We use this period to benchmark changes in attitudes as it was during this time when national lockdowns were implemented, and anxieties were likely at their peak [21, 22]. Here, we conceive of one “treatment” group as those that responded to the survey during lockdown (from March 26, 2020 to April 27th, 2020). The contrasting “control” group consists of participants who responded prior to this period during NZAVS Time 11 (Nov 29, 2019—March 25, 2020). If prejudice started before lockdowns, then the lockdown exposure would appear weaker. As such, the present study reports what would seem to be a "worst case" scenario for pandemic-induced prejudice. Because sampling may not be entirely random with respect to COVID-19 pandemic lockdowns, we attempted to ensure balance in the covariates that might affect prejudice by combining a propensity score model and regression model to achieve doubly-robust causal effect estimation [23]. That is, we use baseline data on the same participants measured in the wave before the pandemic, NZAVS Time 10 (2018–2019), to ensure balance for the covariates in each treatment condition during the COVID-19 wave, NZAVS Time 11 (2019–2020). For a detailed flowchart of the exclusionary criteria, see **Fig 1**.

### Participants and cohort design

#### Eligibility criteria

Individuals who participated in the NZAVS during both Time 10 (the baseline wave) and Time 11 (the treatment wave) were eligible for the study. We include data from Time 10 (June 18, 2020—Nov 29, 2019) to obtain data on baseline covariates to ensure that they were unaffected by the “treatment”, which occurred during Time 11. It is essential to causal inference that no control variables may be affected by treatment condition–an absolute injunction that is notably absent from many experimental studies(see: [23]). We used responses from Time 11 (Nov 20, 2019—Oct 17, 2020) to obtain attitudinal data relevant to infer the effects of prejudice between the treatment groups. Participants were excluded if they responded after lockdowns were lifted on April 27th, 2020, during Time 11. All participants after that point would have experienced the success of New Zealand’s first stringent lockdown, making it difficult to interpret the prejudice effect after that interval because community transmission had been eliminated.

### Analysis

Our causal estimand is the difference in the expected average prejudice had everyone received the COVID-19 lockdown contrasted with the expected average prejudice were everyone measured before lockdown.

It is important to clarify that we cannot use “within-participant” responses from the previous wave as controls for post-treatment outcomes because individuals’ attitudes can change over time [24]. Thus, to obtain valid contrasts, we assessed prejudice in the months immediately preceding the lockdown among the ‘untreated’ group. Minimizing time elapsed between the groups of comparison strengthens causal inference assumptions by restricting time-varying differences between them (see: [25]). Thus, comparisons between the population randomly assigned to the pre-pandemic/post-pandemic “treatments”, controlling for prejudice responses and a host of demographic indicators from the previous year, provides an unbiased estimate for the average treatment effect—or marginal effect—of the pandemic on outcome-wide minority group prejudice. We note that because our sample is restricted to the New Zealand population, our results do not necessarily transport to other populations.

To handle bias from non-response we multiply imputed missing responses using chained equations with the Mice package in R [26]. Prior to imputation, approximately 1.9% of survey responses were incomplete (for missingness of each variable, see **S7 Appendix**). We multiply imputed 10 datasets over which our statistical models averaged results.

Following the protocols described by Greifer, [27] we compared the performance of different weighting methods for achieving balance. We selected the entropy approach from the Weightit package in R because it performed best [28]. **S6 Appendix** reports the standardized difference in means of covariates between conditions before weighting (unadjusted) and after weighting (adjusted). We adopted a threshold of .05 for standardized mean differences (in continuous variables) and differences in proportion (for binary variables). Following Greifer, [27] we adopted a variance ratio threshold between 0.5 and 2. As shown in **S6 Appendix**, all balancing scores (Mean.Diff.Adj) were considerably below the 0.05 threshold and the variance ratio thresholds were all close to 1.

Prior to weighting, the sample size was 28,679 in the untreated (pre-lockdown) and 1,648 in the treated (lockdown). After weighting, the effective sample sizes were 28673.74 in the untreated and 1554.39 in the treated. The most extreme weight in the treated was 2.2701, and it was 1.0601 in the untreated. As expected, these results confirm that features of NZAVS data collection were mostly independent of the timing of the COVID-19 pandemic, but that there may also have been slight selection bias on the sample during lockdown.

We simulated potential outcomes for each individual under each condition (pre-lockdown, lockdown) by combining both observed and predicted outcomes for each participant using their covariates and propensity score weights. That is, we fit a weighted generalized least squares regression model with a Gaussian distribution to estimate prejudice conditional on lockdown condition and covariates. This method was applied to all participants in both counterfactual scenarios, regardless of which condition participants were originally in. Following Griefer et al., [27] we calculated confidence intervals and standard errors using simulation-based inference and robust estimation of error terms. As part of estimation, New Zealand Census weights for Age, Gender, and Ethnicity were added to more closely approximate the country’s population-wide response [29].

We reported the robustness of effects to potential unmeasured confounding using E-values [30]. The E-value is a sensitivity analysis that describes the minimum strength of association an unmeasured confounder would need to have with both the exposure and outcome, conditional on covariates, to explain away the association between the exposure and outcome [30].

Following the outcome-wide approach, [31] we reported effects on all outcomes (12 groups) of which data were available. We used the `margot`package to report and graph results [32].

All analyses were conducted in the R statistical software [33]. Packages used also include dplyr, [34] naniar, [35] table1, [36] mice, [26] here, [37] arrow, [38] visdat, [39] haven, [40] skimr, [41] MatchThem, [42] MatchIt, [43] optmatch, [44] clarify, [45] and cobalt [46].

### Measures

#### Outcomes

The NZAVS measures attitudes toward social groups using feeling thermometers. Feeling thermometers were introduced by the American National Election Study to measure attitudes in terms of “warmth”, where higher “warmth” indicates higher favorability [47]. In the NZAVS, attitudes are measured on a warmth scale ranging from 1 (least warm) to 7 (most warm). We utilize feeling thermometer data toward all 12 groups measured during both the baseline wave and the treatment wave. These groups were: NZ Europeans, Māori, Asians, Pacific peoples, the elderly, refugees, the overweight, immigrants, Chinese, Indians, Muslims, and the mentally ill. To see how feeling thermometers appear in the NZAVS questionnaire, see **S5 Appendix**.

### Covariates

As mentioned, although the occurrence of COVID-19 and New Zealand’s severe lockdown would appear random with respect to NZAVS data collection, it is possible that responses received later in the treatment wave reflected differences in the population, thus leading to imbalance in the covariates that might affect prejudice, perhaps in interaction with the pandemic. To address this possibility, our statistical models (both the outcome regression and propensity score model) included the following covariates as part of a strategy for improving confounding control: ethnicity, age, gender, wealth (New Zealand Deprivation Index), occupation (New Zealand Socioeconomic Index), education, rural area, sub-region, personality (IPIP-6), employed, born in New Zealand, political orientation, parental status, and religious identification. Following Lin et al. [48] and VanderWeele et al., [31] we also included baseline outcome measures for warmth to all social groups (see: **S6 Appendix**). This approach is powerful because it provides causal effect estimates for the incidence of new prejudice over and above baseline levels from the preceding year. Additionally, following the recommendations by Lin et al. [48] and Greifer [28, 45] we included an interaction term of the exposure variable (the pre-pandemic vs. lockdown exposure) and pre-treatment baseline indicators in the outcome regression model. This allowed for adjustment in estimation of interaction between the treatment-effect modifiers and the treatment, thus improving marginal effect estimation for the population.

## Results

Basic demographic information obtained from the baseline wave (NZAVS Time 10) are reported in **Table 1** (for comprehensive participant characteristics, see **S4 Appendix**). At baseline, 63.8% of participants identified as female, 64% identified as non-religious, and 82.9% identified as NZ European. The average age was 50.4 years.

**Table 1:** Baseline participant demographics.

	Overall (N = 30,327)
**Age**	
Mean (SD)	50.4 (13.6)
Median [Min, Max]	52.9 [18.1, 95.5]
**Gender**	
Male	10971 (36.2%)
Female	19356 (63.8%)
**Socio Economic Status (low = 10, high = 90)**	
Mean (SD)	54.9 (16.4)
Median [Min, Max]	56.0 [10.0, 90.0]
Missing	271 (0.9%)
**Education**	
Mean (SD)	5.44 (2.72)
Median [Min, Max]	7.00 [0, 10.0]
Missing	227 (0.7%)
**Religious Identification**	
Yes	10520 (34.7%)
No	19414 (64.0%)
Missing	393 (1.3%)
**Ethnicity**	
European	25128 (82.9%)
Māori	3049 (10.1%)
Pacific	631 (2.1%)
Asian	1296 (4.3%)
Missing	223 (0.7%)

Note.

Education level ranges from 0 (no qualification) to 10 (Doctoral Degree)

### Lockdowns and prejudice

**Table 2** presents the results for estimated risk differences in warmth toward groups between treatment conditions and corresponding E-values. Group warmth scores were standardized (Z-scores) so that effect sizes are comparable across outcomes.

**Table 2:** Estimated difference in warmth between treatment conditions.

Outcome	Estimate (95% CI)	E-Value	CI Limit
**Māori Warmth**	-0.00 (-0.04, 0.04)	1.04	1
**NZEuro Warmth**	-0.01 (-0.05, 0.04)	1.08	1
**Pacific Warmth**	-0.03 (-0.07, 0.02)	1.19	1
**Elderly Warmth**	-0.03 (-0.08, 0.01)	1.21	1
**Overweight Warmth**	-0.03 (-0.07, 0.00)	1.21	1
**Mental illness Warmth**	-0.07 (-0.11, -0.03)	1.32	1.18
**Muslim Warmth**	-0.07 (-0.12, -0.03)	1.34	1.21
**Indians Warmth**	-0.08 (-0.12, -0.04)	1.36	1.23
**Asians Warmth**	-0.08 (-0.12, -0.04)	1.37	1.25
**Refugees Warmth**	-0.09 (-0.13, -0.05)	1.39	1.27
**Immigrant Warmth**	-0.10 (-0.14, -0.06)	1.42	1.29
**Chinese Warmth**	-0.11 (-0.15, -0.07)	1.44	1.32

Note.

a. Estimate = Expected differences in warmth between lockdown conditions for the population on the risk-ratio scale in standard deviations (SD).

b. E-Value = the level that unmeasured confounding would have to take to fully explain away the association between the treatment and outcome, conditional on the measured covariates, on the risk ratio scale.

c. ** = indicates that the E-Value limit is above 1.

d. CI Limit = E-values for the confidence interval limit indicates the minimum association unmeasured confounding would need to have with both the treatment and outcome to shift the confidence interval to include the null, conditional on the measured covariates, on the risk ratio scale.

**Fig 2:**
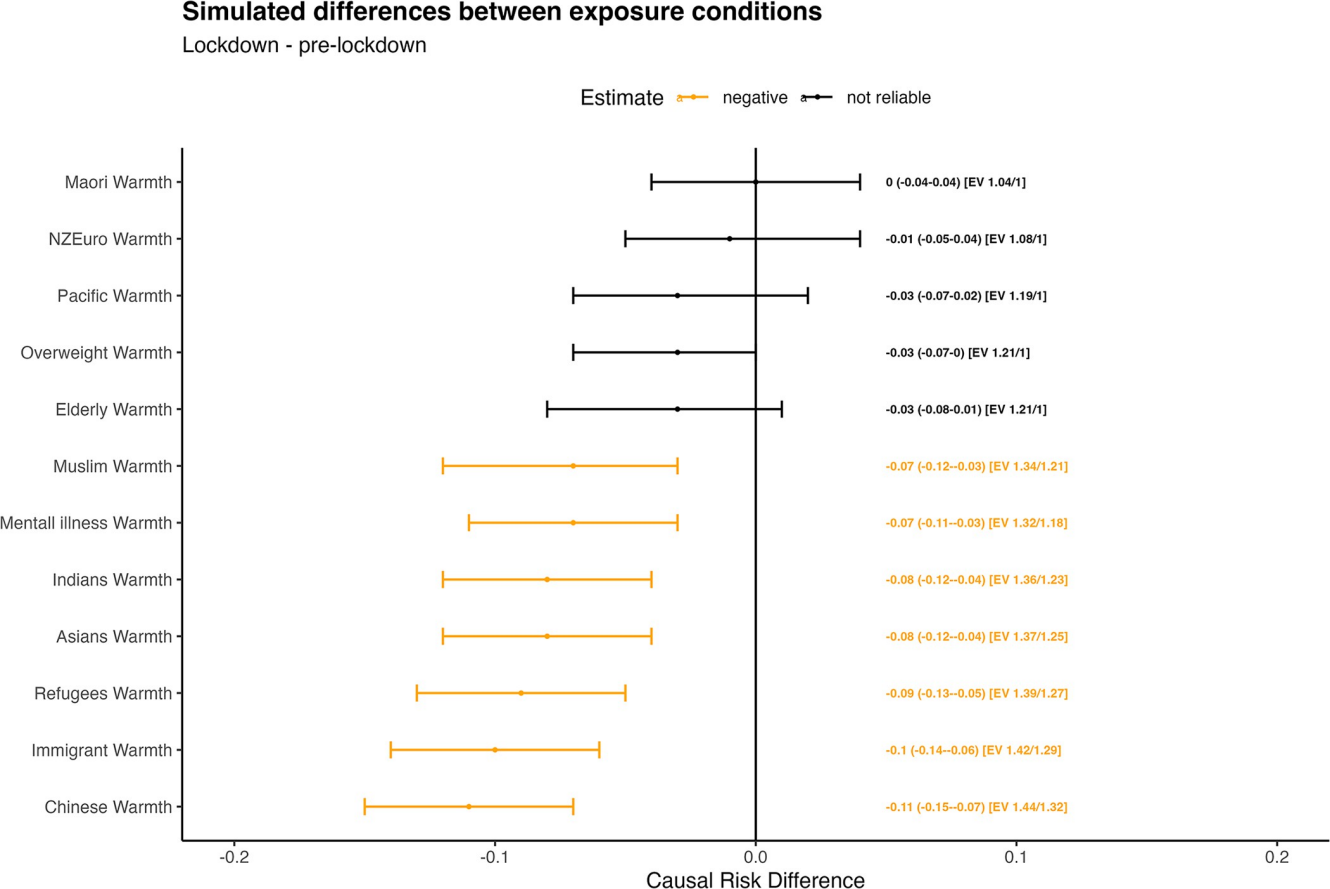
Simulated differences between exposure conditions. Note. a. The causal risk differences are the estimated counterfactual differences in expected average warmth ratings across the population comparing the pandemic treatment and pre-pandemic control conditions. b. Each expected difference in warmth is paired with a point estimate, a 95% confidence interval in parentheses, and respective E-values for the point estimate and lower boundary of the 95% confidence interval in brackets.

Over half of the minority groups measured in the NZAVS experienced reduced warmth during lockdowns, and these effects are robust to a moderate degree of unmeasured confounding. The most reliable evidence for lower warmth was toward Chinese (-0.11 95% CI [-0.15, -0.07]) where unmeasured confounding would need to be associated with both receiving the ‘treatment’ and warmth toward Chinese, conditional on covariates, on the risk ratio scale of at least 1.3 for the confidence interval to include the null. Refugees (-0.09 95% CI [-0.13, -0.05]), Muslims (-0.07 95% CI [-0.12, -0.03]), Asians (-0.08 95% CI [-0.12, -0.04]), immigrants (-0.10 SD, 95% CI [-0.14, -0.06]), and Indians (-0.08 SD, 95% CI [-0.12, -0.04]) experienced lower warmth that is robust to unmeasured confounding, conditional on covariates, of at least 1.2 on the risk ratio scale. Results also indicate evidence for lower warmth toward the mentally ill, though the E-value is lower for this group (1.18). There was no reliable evidence for change in warmth toward Māori (-0.00 SD, 95% CI [-0.04, 0.04]), NZ Europeans (-0.01 SD, 95% CI [-0.05, 0.04]), Pacific peoples (-0.03 SD, 95% CI [-0.07, 0.02]), the elderly (-0.03 SD, 95% CI [-0.08, 0.01]), or the overweight (-0.03 SD, 95% CI [-0.07, 0.00]). The results in **Table 2** are visually displayed for ease of interpretation in **Fig 2**.

## Discussion

### Summary

Previous research suggests that pandemics can induce prejudice. With the discovery of COVID-19 in China, prominent focus has been directed at how social attitudes toward Asians have been affected. However, ample research has documented pandemic prejudice toward other social groups ostensibly unassociated with China. Here, we clarify the question whether anti-Asian prejudice increased during the pandemic across the wider population and whether such prejudice spills over to groups not associated with the virus’ origins. Using panel data to obtain valid baselines for participants before the pandemic, we found that during New Zealand’s first COVID-19 lockdown there were reliable declines in warmth toward Chinese and Asians more broadly, but also toward Refugees, immigrants, Indians, Muslims, and the mentally ill compared with the inferred levels of warmth had the pandemic and lockdown not occurred. By contrast, we do not find evidence for pandemic-induced change in warmth toward Māori, NZ Europeans, Pacific Islanders, the elderly, or the overweight.

Why are certain groups affected and not others? The present study cannot test specific threat-based theories of prejudice, hence, any answer we might provide would be speculative. However, we believe our findings make sense in light of research on national prototypicality, which demonstrates that social groups can be perceived as having varying levels of national belongingness, with some groups seen as more prototypical and defining of the national identity (i.e., ‘us’) and others perceived as more non-prototypical of the national identity or more ‘foreign’. Groups perceived to be more foreign (or non-prototypical) can experience greater prejudice and discrimination under certain conditions, and it may be that pandemics are one of the contexts in which psychological representations of who is part of our national identity is consulted, and we infer greater threat from groups perceived to be more ‘foreign’ (for reviews, see [49, 50]).

Studies show that a prototypical New Zealander is seen as someone who is of European or Māori descent as they are both believed to be representative in the bicultural national identity of New Zealand, while people of Asian descent are perceived as more foreign, even when such individuals are described as being born and raised in New Zealand [50–53]. Further, research on the national character of New Zealand has also included an identification with Pacific Nations culture [54]. Although there is no definitive evidence that Pacific Islanders are considered prototypical, experiments on implicit bias [53] have used facial features of Māori to determine prototypicality, and since Māori originate from Polynesia as other Pacific nation peoples, [55] these results may generalize to include Pacific Island peoples as prototypical. In the context of the current research, not only was the pandemic associated with decreased warmth toward the Chinese and Asians, but also toward other groups who are perceived as relatively ’foreign’ to the national prototype—including Indians (who are also Asian), Muslims (who are associated with the Islamic world and viewed negatively in the context of an exclusive ethnic national prototype [56]), immigrants, and refugees. Broadly, groups perceived as non-prototypical of the national identity may experience a downtick in average warmth during lockdowns.

We note that prejudice toward the mentally ill is an outlier from the perspective of research on national prototypicality, even though this effect was the smallest of all observed. Our natural-experiment method estimates the net effect on prejudicial attitudes and does not distinguish between the effects of specific causes. Hence, which features of the pandemic explain our results, as well as the mechanism of action, remains unclear. Prevailing anxieties during the pandemic lockdowns may have interacted with prior beliefs about individuals with mental illness to promote socially exclusive attitudes. Speculating, the behavior of the mentally ill might have been judged unsafe, and capable of increasing disease vectors from non-compliance with social distancing or poor hygiene. Indeed, some mental illnesses are perceived to be more dangerous or unpredictable, which can lead to social rejection [57, 58]. Future work may seek to identify the causal underpinnings of pandemic prejudice toward the mentally ill.

Again, we cannot test the hypothesis that New Zealanders used national prototypicality as a benchmark to gauge the risk posed by social groups during lockdowns, so the present connection is speculative. As the finding of prejudice toward people with mental illness shows, it is likely that multiple perspectives may explain pandemic-induced prejudice.

### Practical implications

We close by considering two practical implications of our findings.

First, in light of the literature on changing social attitudes, effect sizes generally considered “small” by the standards of psychological research can have a significant impact on affected communities. As we utilize an outcome-wide approach [31] to evaluate the effects on attitudes toward all social groups using standardized units, we can see from Table 2 that none of the outcomes reaches the threshold of 0.2 that is often used to indicate a small effect. Similarly, in prior work, we found that the effects of the Christchurch terrorist attacks, an event of considerable national significance in New Zealand, only elevated Muslim acceptance by well under 0.3 standard deviation units [25]. However, when placed in the context of the background trend of gradually rising Muslim acceptance, this represented about a five-year boost to acceptance in one year [25]. Effects that may appear small outside the tightly controlled settings of randomized experiments can sometimes yield practically significant effects in observational settings–where they make tangible differences in outcomes like hiring decisions, harassment of children at school, tenancy applications, and partnership appointments [59].

Second, we note that against the backdrop of rising acceptance for all social groups, [24] the effects of COVID-19 partially reversed this trend for non-prototypical New Zealand groups. That we detect such effects is concerning and confirms work in other countries that places inter-group prejudice among the hazards of pandemics. However, the practical effects of the prejudice we detect, as well as the duration of these effects, remains unclear.

Thus, our results show evidence of strained intergroup attitudes; but the extent that people were affected by these attitudes remains an open area of inquiry. Follow-up research may investigate the impact of a change in attitudes in a population on the perceived levels of acceptance among affected groups.

### Limitations of the present research

As with any study, ours has its limitations. We highlight two.

First, it is important to acknowledge that the behavioral effects on affected minorities remain speculative. Our aim here has been to document attitudinal effects, the groups for whom they occur, and their magnitude as measured in survey reporting. The extent that behaviors were affected is beyond the scope of this study. Future work must investigate how these effects manifest in social behaviors.

Second, it is unclear whether these effects are long-lasting. In other research, we have found that the impacts of nationally consequential events can endure for several years [25]. However, when attempting to compute effect magnitudes against background trends, we must rely on much stronger assumptions when projecting from the pre-event trend, as we lack direct access to a counterfactual world in which the key event did not occur. Speculating about the long-term effects of COVID-19 on prejudices would require considerable additional assumptions and theory (for a discussion about these issues, see [25]). Given the exploratory nature of this study, we do not attempt to recover long-term trajectories here.

## Conclusion

As this study has shown, psychological panel data with attitudes toward an array of social groups can provide valuable insight into the development of prejudice during epidemics. It is of interest that attitudes toward specific social groups were reliably influenced while others were not, suggesting that New Zealanders calibrated their attitudes toward social groups discriminately in response to the challenges posed at the time. It may be that such reactions were aimed at groups particularly associated with ’foreign’ as the country focused on a shared national identity at the early stages of the pandemic.

These results highlight the need for investigation into the effects of national events and calamities on prejudicial attitudes with causal methods and longitudinal data. Observational research has indicated that distressing events can increase social divisions as well as strengthen solidarity; however, conclusions are typically limited to indeterminate "associations" due to lack of causal framework. If such frameworks were employed, comparative longitudinal studies could complement our findings by examining categories of prototypicality in diverse national contexts and evaluating the influence of effect modifiers of prejudice.

## Supporting information

S1 Appendix Sampling procedure for Time 10. (DOCX)

S2 Appendix Sampling procedure for Time 11. (DOCX)

S3 Appendix Extended COVID-19 timeline. (DOCX)

S4 Appendix Extended participant characteristics at baseline. (DOCX)

S5 Appendix NZAVS feeling thermometer. (DOCX)

S6 Appendix Covariate balance. (DOCX)

S7 Appendix Variable missingness. (DOCX)
