# Supplementary material for: The anatomy of prejudice during pandemic lockdowns: Evidence from a national panel study
Source: PLoS One. 2024 May 28;19(5):e0303845. doi: 10.1371/journal.pone.0303845 (PMC11132491; doi:10.1371/journal.pone.0303845)
Supplement: S1 Appendix — (DOCX) [file pone.0303845.s001.docx]

**Appendix 1**

Sampling Procedure

The Time 10 (2018/2019) NZAVS ran from 18-June-2018 to 30-Nov-2019 and contained responses from 47,948 participants (18,027 retained from one or more previous wave, 29,921 new additions from booster sampling or opt-ins). The sample retained 2,968 participants from the Time 1 (2009/2010) sample (a retention rate of 45.54%). The sample retained 14,050 participants from Time 9 (2017/2018; a retention rate of 82.30% from the previous year). Participants who provided an email address were first emailed and invited to complete an online version if they preferred. Participants who did not complete the online version (or did not provide an email) were then posted a copy of the questionnaire, with a second postal follow-up two months later. We staggered the time of contact, so that participants who had completed the previous wave were contacted approximately one year after they last completed the questionnaire. We offered a prize draw for participation (five draws each for $1000 grocery vouchers, $5000 total prize pool). All participants were posted a Season’s Greetings card from the NZAVS research team and informed that they had been automatically entered into a bonus seasonal grocery voucher prize draw. Participants were also emailed an eight-page newsletter about the study.

To boost sample size and increase sample diversity for subsequent waves, a booster sample was conducted by selecting people from the New Zealand electoral roll. As with previous booster samples, sampling was conducted without replacement (i.e., people included in previous sample frames were identified and removed from the 2018 roll). The sample frame consisted of 325,000 people aged from 18-65 randomly selected from the 2018 New Zealand electoral roll, who were currently residing in New Zealand (one can be registered to vote in New Zealand but living overseas). The electoral roll contained ~3,250,000 registered voters. The New Zealand electoral roll contains participants’ date of birth (within a one-year window), and we limited our frame to people who 65 or younger, due to our aim of retaining participants longitudinally. We concurrently advertised the survey on Facebook via a $5000 paid promotion of a link to a YouTube video describing the NZAVS and the large booster sample we were conducting. The advertisement targeted men and women aged 18-65+ who lived in New Zealand and ran for 14 days. This paid promotion reached 147,296 people, with 4,721 link clicks (i.e., clicking to watch the video), according to Facebook. The goal of the paid promotion was twofold: (a) to increase name recognition of the NZAVS during the period in which questionnaires were being posted, and (b) to help improve retention by potentially reaching previous participants who happened to see the advertisement. A total of 29,293 participants who were contained in our sample frame completed the questionnaire (response rate = 9.20% when adjusting for the 98.20% accuracy of the 2018 electoral roll). A further 628 participants completed the questionnaire, but were unable to be matched to our sample frame (for example, due to a lack of contact information) or were opt-ins. Informal analysis indicates that opt-ins were often the partners of existing participants.

Participants

The Time 10 wave included 29,958 women, 17,783 men, and 207 gender diverse people; with a mean age of 49.10 years (SD = 13.86). With regard to ethnicity, 42,543 people identified as European, 4,696 as Māori, 1,039 were Pacific Nations peoples, and 2,541 identified with an Asian ethnic group. Note that people could identify with multiple ethnic groups (and hence be counted multiple times). Education (M = 5.32, SD = 2.73) was coded using the New Zealand Qualifications Authority scheme, which ranged from 0 (none) to 10 (doctoral degree or equivalent). Deprivation (M = 4.77, SD = 2.73) was coded using the New Zealand Deprivation index for the meshblock level (approx. 100 person-sized geographic units), with a decile rank from 1 (low) 10 (high; Atkinson, Salmond & Crampton, 2014). Socioeconomic status (M = 54.47, SD = 16.59) was scored from 10 (low) to 90 (high) using the New Zealand Socioeconomic Index, which assigned a score based on occupation and derived from census data (Fahy, Lee, & Milne, 2017). Mean household income was NZ$ 115,061 (SD = NZ$ 92,445, median = NZ$ 100,000). Of the 47,948 people sampled, 17,141 were religious; 33,631 were parents; 34,218 had a romantic partner; 38,024 were employed; and 37,126 were born in New Zealand.


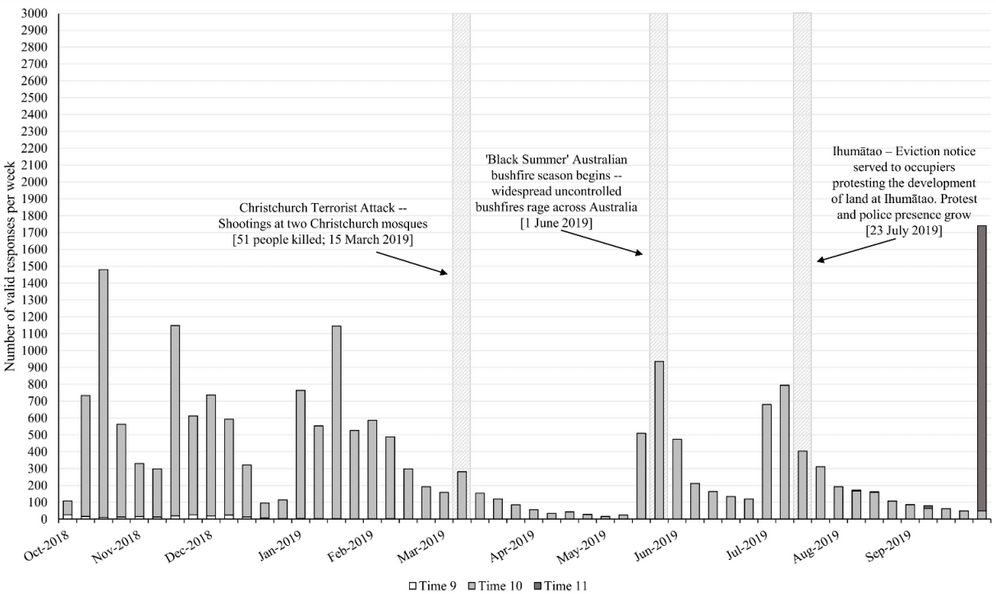


Figure 10. Histogram of weekly survey completions for Time 10 conducted from 18-June-2018 to 30.-Nov-2019 (x axis ranges from October 2018 to October 2019, some responses to this wave may be presented on the graph for the following year; select national and international events that occurred during this data collection wave are also included for context).
