# Supplementary material for: The anatomy of prejudice during pandemic lockdowns: Evidence from a national panel study
Source: PLoS One. 2024 May 28;19(5):e0303845. doi: 10.1371/journal.pone.0303845 (PMC11132491; doi:10.1371/journal.pone.0303845)
Supplement: S2 Appendix — (DOCX) [file pone.0303845.s002.docx]

**Appendix 2**

Sampling Procedure

The Time 11 wave was conducted during COVID-19 pandemic, and ran from 29-Nov-2019 to 17-Oct-2020. Procedures thus differed in that there was an increased focus on online deliver using email reminders and extensive Facebook advertising, no Christmas card, and incomplete phoning of non-respondents.

The Time 11 (2019/2020) NZAVS contained responses from 42,681 participants (36,527 retained from one or more previous wave, 6,154 new additions from booster sampling or opt-ins). The sample retained 2,506 participants from the Time 1 (2009/2010) sample (a retention rate of 38.45%). The sample retained 34,783 participants from Time 10 (2018; a retention rate of 72.54% from the previous year). Participants who provided an email address were first emailed and invited to complete an online version if they preferred. Participants who did not complete the online version (or did not provide an email) were then posted a copy of the questionnaire, with a second postal follow-up two months later. We staggered the time of contact, so that participants who had completed the previous wave were contacted approximately one year after they last completed the questionnaire. A second reminder email was sent approximately four months following the first email attempt. We offered a prize draw for participation (five draws each for $1000 grocery vouchers, $5000 total prize pool). Participants were also emailed an eight-page newsletter about the study. As in past years, three attempts were made to phone non-respondents using each available cell and landline number. However, due to the university closure during COVID-19 lockdowns, phoning attempts were made for only 54.00% of the phoning pool (11,687 from a total of 21,636 non-respondents who provided at least one phone number).

Two additional forms of recruitment were also introduced during Time 11. The first was a large information box in the questionnaire (taking a full page on the paper version), which asked people: ‘Do you have a partner who would also like to join the NZAVS?’ with additional details for how partners might join the study (see questionnaire for the full text). The second was a Facebook/Instagram advertisement. The advertisement targeted men and women aged 18-65+ who lived in New Zealand and ran from and 4thApril 2020 – 4th July 2020 (overlapping with New Zeeland’s first lockdown period and recovery), and again from 18th August 2020 – 4thSeptember (during the second Auckland lockdown). Given the unprecedented nature of the COVID-19 lockdowns, we thought it important to maximise sampling during these periods. The goal of the Facebook/Instagram advertisement was threefold: (a) to increase name recognition of the NZAVS and remind people to complete the paper/online version already posted/emailed to them, (b) to help improve retention by potentially reaching previous lost participants who happened to see the advertisement, and (c) to recruit new participants (and also the partners of existing participants) while people were at home with some possibly having more free time during lockdown. This last goal was indirect and not explicitly stated it in the advertisement.

The Facebook/Instagram advertisement read as follows: “Participate in the New Zealand Attitudes and Values Study. Complete the 2020 Questionnaire online” with the body of text: “If you are part of the NZAVS, but have not heard from us in the last year, then please consider completing the 2020 questionnaire online. The study is more important than ever as we aim to understand the impact of COVID-19 on mental health, wellbeing and resilience in our communities. Note that the advertisement referred to the year 2020 as this was conducted early in that year, as part of the Time 11 (2019 wave. We wish you all the best at this time and hope you keep well and stay safe.” This paid promotion reached 883,969 people, with 37,850 link clicks (i.e., clicking the link for the Qualtrics survey) according to Facebook. A total of 6,106 people continued complete the questionnaire and provide full contact details, and were thus included in the dataset (4,734 were new participants opting in to the study, and 1,372 were previously ‘lost’ participants).

Participants

The Time 11 wave included 27,176 women, 15,238 men, and 269 gender diverse people, with a mean age of 52.05 years (SD = 13.87). With regard to ethnicity, 39,525 people identified as European, 4,314 as Māori, 1,148 were Pacific Nations peoples, and 1900 identified with an Asian ethnic group. Note that people could identify with multiple ethnic groups (and hence be counted multiple times). Education (M = 5.66, SD = 2.67) was coded using the New Zealand Qualifications Authority scheme, which ranged from 0 (none) to 10 (doctoral degree or equivalent). Deprivation (M = 4.75, SD = 2.72) was coded using the New Zealand Deprivation index for the meshblock level (approx. 100 person-sized geographic units), with a decile rank from 1 (low) 10 (high; Atkinson, Salmond & Crampton, 2014). Socioeconomic status (M = 59.93, SD = 16.19) was scored from 10 (low) to 90 (high) using the New Zealand Socioeconomic Index, which assigned a score based on occupation and derived from census data (Fahy, Lee, & Milne, 2017). Mean household income was NZ$ 117,971 (SD = NZ$ 109,738, median = NZ$ 100,000). Of the 42,681 people sampled, 14,375 were religious; 31,213 were parents; 31,107 had a romantic partner; 31,856 were employed; and 33,367 were born in New Zealand.


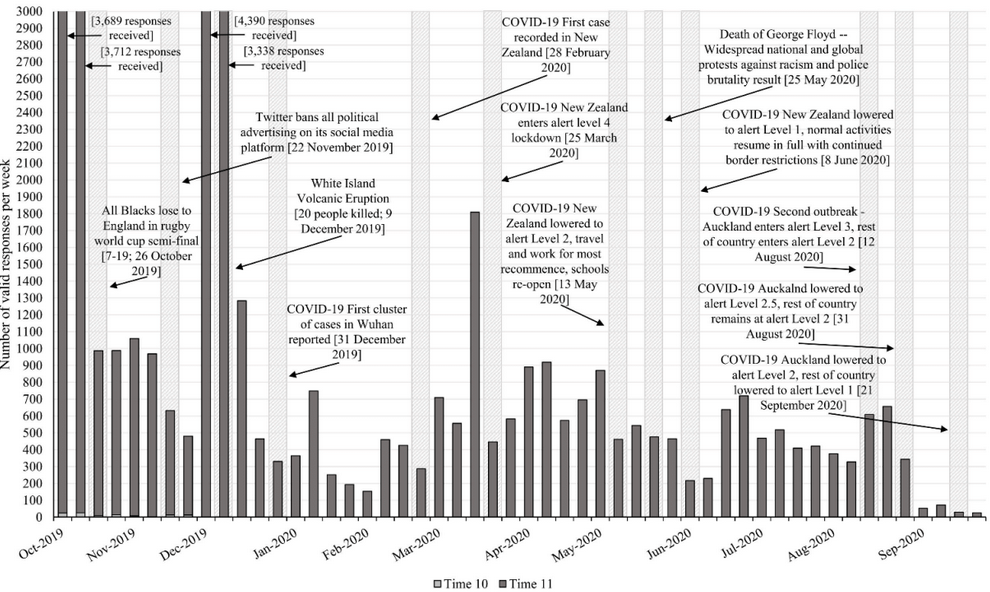


Figure 11. Histogram of weekly survey completions for Time 11 (x axis ranges from October 2019 to October 2020, some responses to this wave may be presented on the graph for the following year; select national and international events that occurred during this data collection wave are also included for context).
