# Supplementary material for: The anatomy of prejudice during pandemic lockdowns: Evidence from a national panel study
Source: PLoS One. 2024 May 28;19(5):e0303845. doi: 10.1371/journal.pone.0303845 (PMC11132491; doi:10.1371/journal.pone.0303845)
Supplement: S3 Appendix — (DOCX) [file pone.0303845.s003.docx]

**Appendix 3**

On December 31, 2019, The World Health Organization (WHO) was informed about a viral disease outbreak in China.(1) As the disease began to spread internationally, the WHO declared a public health emergency on January 31, 2020.(2) But it was not until February 28 that the first case of the new disease, COVID-19, was reported in New Zealand.(2) In the following weeks, confirmed cases continued to climb. By March 19, the total had risen to 28, but community transmission was not yet a concern, as the Director-General of Health Ashley Bloomfield said all cases were connected to international travel.(1) The same day, New Zealand closed its borders to all but citizens and permanent residents.(2) On March 21, there were 52 confirmed cases of COVID-19 in the country, but the government was not able to rule out community transmission for two.(1) On March 23, the two cases were officially considered community transmission, and the government announced heightened restrictions would take place immediately with lockdowns taking place in 48 hours.(1) Then, a state of national emergency was declared on March 25 and stringent country-wide lockdowns were implemented at 11:59 pm. (2) During lockdown, New Zealand reported its first COVID-19 related death.(2) After over a month of country-wide quarantine, the lockdowns were lifted and replaced with progressively less stringent restrictions starting on April 27^th^.(2) By May 4, New Zealand did not have any new confirmed cases of the disease.(2)

1. Strongman S. Covid-19 pandemic timeline [Internet]. [cited 2024 Feb 8]. Available from: http://shorthand.radionz.co.nz/coronavirus-timeline/index.html

2. Unite against COVID-19 [Internet]. 2022 [cited 2024 Feb 5]. History of the COVID-19 Alert System. Available from: https://covid19.govt.nz/about-our-covid-19-response/history-of-the-covid-19-alert-system/
