# Supplementary material for: The anatomy of prejudice during pandemic lockdowns: Evidence from a national panel study
Source: PLoS One. 2024 May 28;19(5):e0303845. doi: 10.1371/journal.pone.0303845 (PMC11132491; doi:10.1371/journal.pone.0303845)
Supplement: S4 Appendix — (DOCX) [file pone.0303845.s004.docx]

#### **Appendix 4**

|  | Overall |
| --- | --- |
|  | (N=30327) |
| **Ethnicity** |  |
| European | 25128 (82.9%) |
| Maori | 3049 (10.1%) |
| Pacific | 631 (2.1%) |
| Asian | 1296 (4.3%) |
| Missing | 223 (0.7%) |
| **Age** |  |
| Mean (SD) | 50.4 (13.6) |
| Median [Min, Max] | 52.9 [18.1, 95.5] |
| **Gender** |  |
| Male | 10971 (36.2%) |
| Female | 19356 (63.8%) |
| **Deprivation** **Index** (low = 1, high = 10) |  |
| Mean (SD) | 4.68 (2.70) |
| Median [Min, Max] | 4.00 [1.00, 10.0] |
| Missing | 222 (0.7%) |
| **Urban** **Area** |  |
| High Urban Accessibility | 18578 (61.3%) |
| Medium Urban Accessibility | 5820 (19.2%) |
| Low Urban Accessibility | 3696 (12.2%) |
| Remote | 1658 (5.5%) |
| Very Remote | 355 (1.2%) |
| Missing | 220 (0.7%) |
| **Socio** **Economic** **Status** (low = 10, high = 90) |  |
| Mean (SD) | 54.9 (16.4) |
| Median [Min, Max] | 56.0 [10.0, 90.0] |
| Missing | 271 (0.9%) |
| **Education** |  |
| Mean (SD) | 5.44 (2.72) |
| Median [Min, Max] | 7.00 [0, 10.0] |
| Missing | 227 (0.7%) |
| **Region** |  |
| Northland Region | 1034 (3.4%) |
| Auckland Region | 8052 (26.6%) |
| Waikato Region | 2502 (8.3%) |
| Bay of Plenty Region | 1831 (6.0%) |
| Gisborne Region | 209 (0.7%) |
| Hawkes Bay Region | 1086 (3.6%) |
| Taranaki Region | 756 (2.5%) |
| Manawatu-Whanganui Region | 1569 (5.2%) |
| Wellington Region | 4604 (15.2%) |
| West Coast Region | 249 (0.8%) |
| Canterbury Region | 4417 (14.6%) |
| Otago Region | 1825 (6.0%) |
| Southland Region | 640 (2.1%) |
| Tasman Region | 461 (1.5%) |
| Nelson Region | 481 (1.6%) |
| Marlborough Region | 387 (1.3%) |
| Area Outside Region | 4 (0.0%) |
| Missing | 220 (0.7%) |
| **Conscientiousness** |  |
| Mean (SD) | 5.15 (1.05) |
| Median [Min, Max] | 5.25 [1.00, 7.00] |
| Missing | 269 (0.9%) |
| **Openness** |  |
| Mean (SD) | 4.98 (1.12) |
| Median [Min, Max] | 5.00 [1.00, 7.00] |
| Missing | 270 (0.9%) |
| **Honesty/Humility** |  |
| Mean (SD) | 5.49 (1.15) |
| Median [Min, Max] | 5.75 [1.00, 7.00] |
| Missing | 273 (0.9%) |
| **Extraversion** |  |
| Mean (SD) | 3.87 (1.20) |
| Median [Min, Max] | 3.75 [1.00, 7.00] |
| Missing | 269 (0.9%) |
| **Neuroticism** |  |
| Mean (SD) | 3.45 (1.16) |
| Median [Min, Max] | 3.50 [1.00, 7.00] |
| Missing | 277 (0.9%) |
| **Agreeableness** |  |
| Mean (SD) | 5.37 (0.983) |
| Median [Min, Max] | 5.50 [1.00, 7.00] |
| Missing | 275 (0.9%) |
| Employed |  |
| Employed.1 | 24036 (79.3%) |
| Unemployed | 6244 (20.6%) |
| Missing | 47 (0.2%) |
| **Born in NZ** |  |
| Yes | 24002 (79.1%) |
| No | 6309 (20.8%) |
| Missing | 16 (0.1%) |
| **Political Orientation** |  |
| Mean (SD) | 3.55 (1.39) |
| Median [Min, Max] | 4.00 [1.00, 7.00] |
| Missing | 1811 (6.0%) |
| **Parent** |  |
| Yes | 21572 (71.1%) |
| No | 8743 (28.8%) |
| Missing | 12 (0.0%) |
| **Religious Identification** |  |
| Yes | 10520 (34.7%) |
| No | 19414 (64.0%) |
| Missing | 393 (1.3%) |

Note.

1. As covariates can be influenced by the exposure, we report covariates taken from baseline (NZAVS data collection wave 10 (2018-2019)).
2. Education level ranges from 0 (no qualification) to 10 (doctoral degree).
3. Political orientation scale is from 1 (very liberal) to 7 (very conservative).
4. Personality variables (conscientiousness, openness, honesty/humility, extraversion, neuroticism, and agreeableness) are on scale from 1 (low) to 7 (high).
5. Deprivation Index = neighborhood socioeconomic level.
