# Supplementary material for: The anatomy of prejudice during pandemic lockdowns: Evidence from a national panel study
Source: PLoS One. 2024 May 28;19(5):e0303845. doi: 10.1371/journal.pone.0303845 (PMC11132491; doi:10.1371/journal.pone.0303845)
Supplement: S5 Appendix — (DOCX) [file pone.0303845.s005.docx]

**Appendix 5**


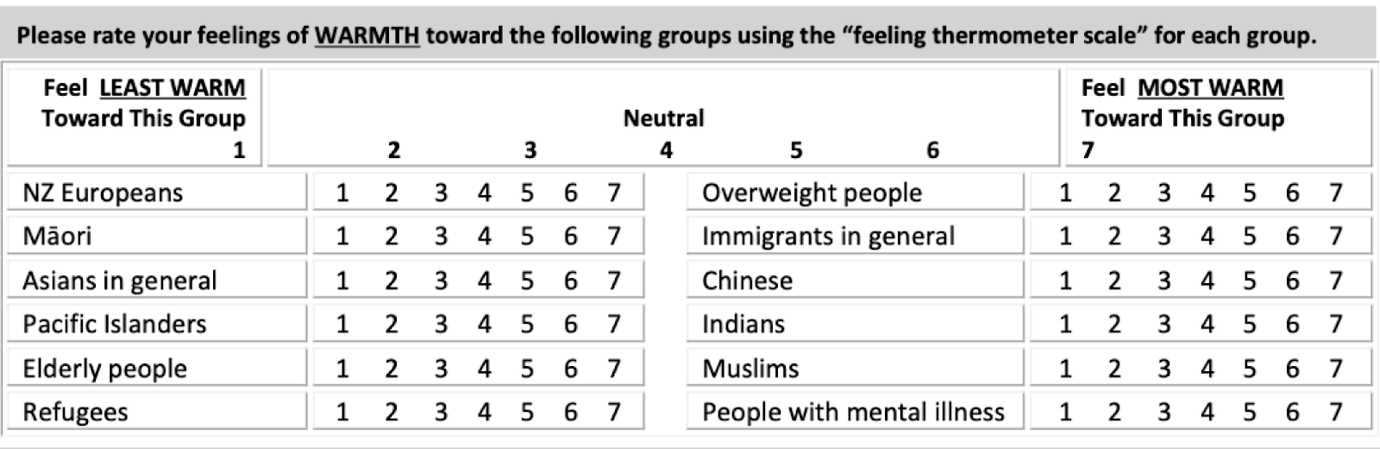


Note.

1. Group warmth is measured through a feeling thermometer in the New Zealand Attitudes and Values Study (NZAVS) dataset.
