# Supplementary material for: The anatomy of prejudice during pandemic lockdowns: Evidence from a national panel study
Source: PLoS One. 2024 May 28;19(5):e0303845. doi: 10.1371/journal.pone.0303845 (PMC11132491; doi:10.1371/journal.pone.0303845)
Supplement: S6 Appendix — (DOCX) [file pone.0303845.s006.docx]

## Appendix 6

|  | Mean.Diff.Adj | Mean.V.Ratio.Adj |
| --- | --- | --- |
| t0_EthCat | 0.0000399 | 0.9081100 |
| t0_Rural_GCH2018 | 0.0000228 | 0.9833192 |
| t0_REGC_2022 | 0.0000356 | 0.9499717 |
| t0_Partner_z | 0.0000592 | 1.0005380 |
| t0_Age_z | -0.0000192 | 1.0061164 |
| t0_Male_z | 0.0000242 | 1.0006227 |
| t0_NZSEI13_z | -0.0000503 | 0.9662061 |
| t0_NZDep2018_z | 0.0000492 | 1.0035055 |
| t0_CONSCIENTIOUSNESS_z | 0.0000006 | 1.0275306 |
| t0_OPENNESS_z | -0.0000618 | 1.0066885 |
| t0_HONESTY_HUMILITY_z | -0.0000672 | 0.9251611 |
| t0_EXTRAVERSION_z | -0.0000669 | 1.0459074 |
| t0_NEUROTICISM_z | 0.0000191 | 0.9835963 |
| t0_AGREEABLENESS_z | 0.0000113 | 1.0522301 |
| t0_Edu_z | -0.0001496 | 0.9849501 |
| t0_Employed_z | 0.0002082 | 1.0003074 |
| t0_BornNZ_z | -0.0000234 | 1.0006424 |
| t0_Pol.Orient_z | 0.0000664 | 1.0128462 |
| t0_Parent_z | -0.0001879 | 1.0007837 |
| t0_Relid_z | -0.0000301 | 1.0023525 |
| t0_Warm.Muslims_z | -0.0001848 | 0.9816849 |
| t0_Warm.Asians_z | -0.0000502 | 0.9432697 |
| t0_Warm.Chinese_z | -0.0000305 | 0.9801836 |
| t0_Warm.Elderly_z | -0.0000610 | 0.9499461 |
| t0_Warm.Immigrants_z | -0.0000505 | 0.9821058 |
| t0_Warm.Indians_z | -0.0000817 | 0.9910996 |
| t0_Warm.Maori_z | -0.0000049 | 1.0075044 |
| t0_Warm.MentalIllness_z | -0.0000718 | 0.9051495 |
| t0_Warm.NZEuro_z | -0.0000305 | 1.0101863 |
| t0_Warm.Overweight_z | -0.0000399 | 0.9504509 |
| t0_Warm.Pacific_z | -0.0000440 | 0.9655460 |
| t0_Warm.Refugees_z | -0.0000558 | 0.9474871 |

Note

1. t0 = pre-lockdown wave.
2. “_z” = Variable was Z-score transformed.
3. Rural_GCH2018 = Rural/urban area.
4. REGC_2022 = Region.
5. NZSEI13 = Socioeconomic status.
6. NZdep2018 = NZ neighborhood deprivation index.
7. Edu = Education level.
8. BornNZ = Born in New Zealand.
9. Pol.Orient = Political orientation.
10. Parent = Participant is a parent.
11. Relid = Religious Identification.
