# Supplementary material for: The anatomy of prejudice during pandemic lockdowns: Evidence from a national panel study
Source: PLoS One. 2024 May 28;19(5):e0303845. doi: 10.1371/journal.pone.0303845 (PMC11132491; doi:10.1371/journal.pone.0303845)
Supplement: S7 Appendix — (DOCX) [file pone.0303845.s007.docx]

**Appendix 7**


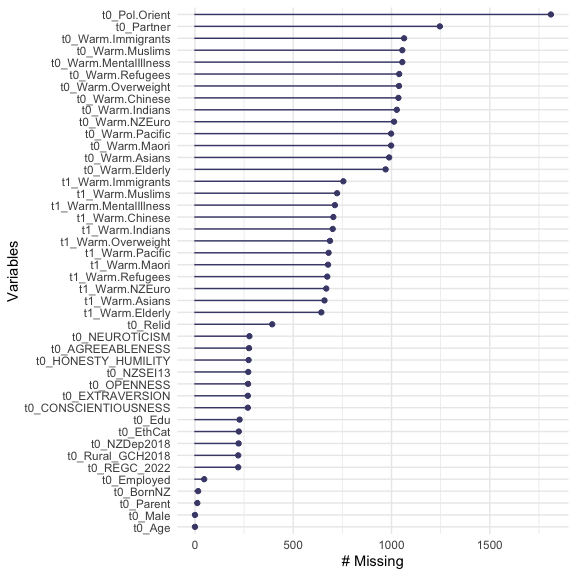


Note.

1. Here, we show the number of missing responses for each variable.
2. t0 = Pre-lockdown wave (Time 10), t1 = Lockdown wave (Time 11).
3. REGC_2022 = Region.
4. NZSEI13 = Socioeconomic status.
5. NZdep2018 = NZ neighborhood deprivation index.
6. Edu = Education.
7. BornNZ = Born in New Zealand.
8. Pol.Orient = Political orientation.
9. Parent = Participant is a parent.
10. Relid = Religious identification.
11. EthCat = Ethnicity.
12. Partner = Relationship status.
13. Male = Gender.
14. Rural_GCH2018 = Rural/urban area.
